# Acouphènes révélateurs de la maladie de Horton

**DOI:** 10.11604/pamj.2015.22.309.7780

**Published:** 2015-11-26

**Authors:** Saloua Ouraini, Ahmed Rouihi, Ismail Nakkabi, Fouad Benariba

**Affiliations:** 1 Service d'ORL et CCF, Hôpital Militaire d'Instruction Mohammed V, CHU Ibn Sina, Rabat, Maroc

**Keywords:** Maladie de Horton, acouphène, corticoïde, Horton disease, Tinnitus, corticoide

## Abstract

La maladie de Horton est une artérite giganto-cellulaire touchant les artères de gros et de moyen calibre du territoire céphalique. Elle a une expression clinico-biologique polymorphe. Le diagnostic de certitude est posé devant des formes typiques comportant des critères diagnostiques cliniques et paracliniques établis par l'ACR (American College of Rhumatology). Cependant certaines formes atypiques de la maladie peuvent faire retarder le diagnostic, c'est le cas notamment des formes révélées par des manifestations ORL qui sont inhabituelles et peu spécifiques. Le but de ce travail est de rapporter un cas atypique de la maladie de Horton révélé par des acouphènes, le retard diagnostique pouvant être à l'origine d'une mauvaise prise en charge thérapeutique, avec des risques de complications vasculaires graves.

## Introduction

La maladie de Horton ou « artérite à cellules géantes » est une entité clinique, radiologique et histologique bien connue dans sa forme typique. Il s'agit d'une affection classique du sujet âgé atteignant avec prédilection les artères temporales et dont la plurifocalité de l'atteinte artérielle explique le polymorphisme clinique; Cependant elle pose un problème de diagnostic devant des signes inauguraux atypiques. Ces formes atypiques méritent d’être bien connues pour permettre un diagnostic précoce et la mise en route de la corticothérapie, afin d’éviter l'apparition des complications graves et irréversibles [1]. La littérature médicale est très riche en cas cliniques faisant état de manifestations exceptionnelles extrêmement diverses, le plus souvent corticosensibles, elles sont pour la plupart liées à une atteinte artérielles distale peu fréquente, elles sont rattachées à la maladie de Horton soit en raison de leur survenue au cours de la maladie, soit en raison du résultat de la biopsie de l'artère temporale (BAT). Les manifestations otologiques atypiques observées dans les maladies systémiques sont très diverses et parfois révélatrices. Une collaboration entre spécialiste ORL, rhumatologues et internistes est indispensable pour poser le diagnostic et éviter les retards diagnostiques.

## Patient et observation

**Figure 1:**
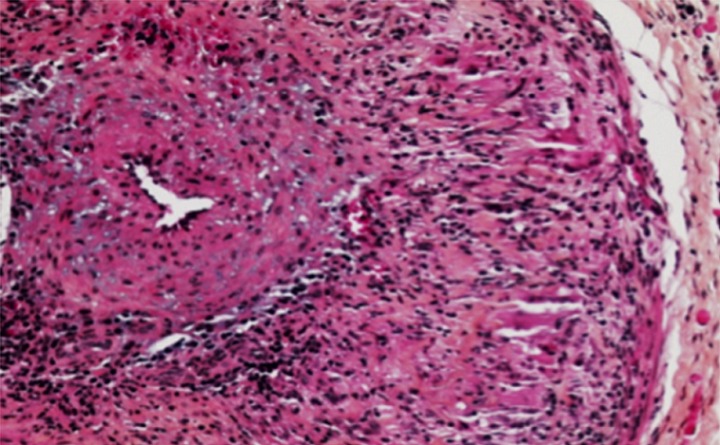
Panartérite granulomateuse

Mr M.A âgé de 64 ans sans antécédents pathologiques notables, a présenté des acouphènes gauches permanents, évoluant depuis 1 semaine, les bilans neurologique et cardiaque étaient normaux et l'examen cochléo-vestibulaire comportant un bilan audiométrique et otoscopique était sans particularité. Le patient a été mis sous traitement symptomatique (vasodilatateur + antalgiques) pendant 4 semaines sans amélioration. L’évolution a été marquée par l'apparition de céphalées temporales et d'un amaigrissement chiffré à 8 kg, un examen clinique général n'a pas identifié de signes articulaires ou d'adénopathies, l'auscultation cardiaque était normale, ainsi que l'examen ophtalmologique; Une pathologie sinusienne, pulmonaire ou digestive a également été recherchée par des scanner thoraco-abdomino-pelvien et sinusien qui sont revenus normaux. Par ailleurs le bilan biologique a retrouvé une CRP à 240 mg/l. Devant ce syndrome inflammatoire associé à des céphalées temporales et à une altération de l’état général chez un sujet âgé, le diagnostic d'une vascularite systémique a été évoqué et une biopsie de l'artère temporale gauche a été réalisée. L’étude anatomopathologique a été faite, ce qui a confirmé le diagnostic de la maladie de Horton en retrouvant une lumière artérielle rétrécie, occupée par un thrombus fibrino-cruorique avec une paroi artérielle inflammatoire, des tuniques pariétales siège d'une fibrose au sein de laquelle il existe un infiltrat inflammatoire lympho-plasmocytaire et histiocytaire avec une limitante élastique interne fragmentée (Figure 1). Une corticothérapie intraveineuse à la dose de 1 mg/kg/jour a été débutée pendant 5 jours relayée par la voie orale pendant 2 semaines, ce qui a permis la normalisation de la CRP avec disparition complète des céphalées et des signes généraux ainsi qu'une régression des acouphènes. Ce résultat obtenu, la posologie a été diminuée progressivement par paliers uniformes hebdomadaires de 10 mg sur 6 semaines jusqu’à 0,35 mg/kg/j. Ensuite la réduction a été de 1mg tous les 10 jours jusqu’à la dose d'entretien de 5 mg/j. L’évolution a été marquée par l'absence de récidive de la symptomatologie initiale. Le recul est de 12 mois.

## Discussion

La maladie de Horton est une artérite giganto-cellulaire ou panarterite à cellules géantes segmentaire et plurifocale touchant les artères de gros et de moyen calibre du territoire céphalique (surtout les branches de la carotide externe dont l'artère temporale) [1–3], mais capable de diffuser à tous les gros troncs artériels. Elle a été décrite pour la première fois par Hutchinson en 1890 et reconnue après sa publication par Horton en 1932. Sur le plan histologique elle se caractérise par un aspect typique de panarterite inflammatoire atteignant les 3 tuniques de l'artère avec un épaississement intimal et fibrose, une infiltration granulomateuse à cellules géantes qui prédomine au sein de la media et rompant la limitante élastique interne [2, 4, 5]. C'est une vascularite qui concerne en général la personne âgée, avec une incidence maximum dans la fourchette des 70 à 75 ans. Elle est exceptionnelle avant l’âge de 50 ans. On note une prévalence féminine [2]. Le début est souvent progressif et d'apparence banale pouvant comporter des céphalées, une altération de l’état général avec fébricule et amaigrissement, mais aussi des signes articulaires avec des arthralgies des ceintures scapulaires ou pelviennes. La forme typique [5, 6] réalise un trépied symptomatique associant des céphalées dans 60% des cas qui touchent généralement le territoire temporal uni ou bilatérale a type de brûlures superficielles souvent pulsatiles et qui sont accentués par l'attouchement, une altération de l’état général (une fois sur deux) avec amaigrissement, fébricule ou fièvre (39-40°C) et des manifestations rhumatismales dans 40 à 50% des cas qui peuvent réalisées un tableau de pseudopolyarthrite rhizomelique avec enraidissement douloureux des épaules associé souvent a des cervicalgies. La ceinture pelvienne peut également être touchée avec des douleurs au niveau de la racine des cuisses. Il peut se voir rarement des arthralgies des grosses articulations périphériques avec des signes inflammatoires locaux. Chacun des éléments de ce trépied peut cependant être isolé, rendant le diagnostic plus difficile, en plus dans 5 à 38% des cas, les symptômes typiques sont minimes ou inexistants. Des critères, établis en 1990 par le Collège Américain de rhumatologie permettent de poser le diagnostic de la maladie de Horton [7, 8]: l’âge supérieur ou égal a 50 ans; les céphalées localisées récente ou modification récente d'une céphalée ancienne; la sensibilité de l'artère temporale a la palpation ou la diminution du pouls temporal; la vitesse de sédimentation supérieure ou égale a 50 mm/h; la biopsie de l'artère temporale anormale, en son absence, l'existence d'un syndrome inflammatoire biologique et une amélioration rapide des symptômes sous corticoïdes sont requis.

La présence de 3 des 5 critères signe la maladie, dont l'histologie fait le diagnostic avec une sensibilité de 93,5% et une spécificité de 91,2%, ainsi une biopsie négative ne remet pas en cause le diagnostic devant une forte conviction clinique. Le diagnostic ne reste difficile que devant des formes atypiques. La mise en évidence de signes inhabituels peut améliorer pour l'avenir la pertinence diagnostique et éviter par la suite l’évolution vers des complications. Notre observation illustre cette difficulté diagnostique de la maladie de Horton puisque l'atteinte otologique avec les acouphènes unilatéraux sont au premier plan sans signes anamnestiques orientant vers cette maladie. Les manifestations ORL spécifiques de la maladie de Horton sont rares, rarement révélatrices, caractérisées par des symptômes banaux, elles peuvent s'associer aux manifestations stomatologiques de la maladie [9, 10] tel que la claudication ischémique de la mâchoire, les douleurs dentaires ou maxillaires, la glossodynie, le trismus, l’œdème jugulaire et/ou de la face, les douleurs pharyngiennes ou laryngées, la dysphagie, la dysphonie, la glossodynie, les otalgies, les vertiges ou la surdité brusque [11]. Les acouphènes sont une manifestation inaugurale très rare, pouvant égarer le diagnostic vers une affection otorhinolaryngologique ou neurologique et faisant retarder le diagnostique. Il s'agit d'acouphènes subjectifs liés à la vascularite [12], engendrés par le syndrome inflammatoire. Leurs évolution est parallèle à celle de la symptomatologie générale de la maladie, elle est généralement régressive sous corticothérapie et la rechute est parfois possible lors de la décroissance ou l'arrêt du traitement. La maladie de Horton est une urgence thérapeutique car une complication vasculaire (ischémie) [5, 7, 13] essentiellement ophtalmologique (cécité), parfois neurologique (accident vasculaire cérébral) ou cardiaque (aortite, coronarite) peut survenir inopinément à tout moment. Cette complication survenait dans 60% des cas avant l’ère des corticoïdes. La corticothérapie est le seul traitement ayant prouvé son efficacité [5, 14], son effet spectaculaire est considéré comme un test diagnostic. Les corticoïdes d'action courte et de bonne biodisponibilité, dont le Prédnisone ou le Méthylprednisone, sont à privilégier. D'autres médicaments [8] (Hydroxychloroquine, Dapsone, Méthotrexate, Aziathioprine, anti-TNF (anti-Tumour Necrosis Factor)) n'ont pas fait la preuve de leur efficacité isolement. Ils peuvent être utilisés en cas d’échec thérapeutique ou d'intolérance majeure aux corticoïdes. La dose initiale de corticoïde doit être forte pour contrôler non seulement l'inflammation artérielle, mais aussi le processus immunologique à son origine. La dose quotidienne de 0,7 à 1mg /kg du patient est administrée dans les formes non compliquées, dans le cas contraire une plus forte dose est nécessaire de l'ordre de 1 mg/kg/j généralement précédée d'un bolus intraveineux pendant 3 jours. Un bon contrôle de la maladie implique la disparition des signes généraux, de toutes les manifestations fonctionnelles réversibles et l'absence d'inflammation biologique (normalisation de la CRP), soit 2 à 3 semaines pour plus de 80% des cas. Une réduction rapide des corticoïdes doit être envisagée afin de contrer leurs effets indesirables, elle est réalisée par palier hebdomadaire étalé sur 4 à 6 semaines jusqu’à 0,35 mg /kg/j. Par la suite la réduction est beaucoup plus lente, elle est de 1 mg toutes les 10 à 15 jours, puis de 1 mg /mois jusqu’à l'arrêt. La majorité des malades sont sevrés en moyenne entre 18 mois à 3 ans [7]. L'adjonction de traitements anticoagulants ou antiagrégants plaquettaire est recommandée dans le but de réduire les risques de complications ischémiques initiales [2]. La cortico-dépendance définie comme la survenue de reprise évolutive lors de la phase de décroissance du traitement ou après son arrêt est fréquente. Elle impose de rechercher une cause intercurrente, surtout une infection latente, avec la nécessité de fixer un objectif: atteindre une dose sécuritaire d'entretien de 5mg/j. La cortico-résistance primaire, définie comme l'impossibilité de contrôler les signes cliniques ou biologiques de la maladie, ou l'impossibilité de diminuer les doses d'attaque sans voire réapparaitre des symptômes, est exceptionnelle. Il pourra être associé dans ce cas un immunosuppresseur comme le Méthotrexate [6]. La surveillance des récidives repose sur la clinique et sur la CRP avec une moyenne de 20 à 30% de récidives, principalement au cours de l'année qui suit, elles peuvent être multiples chez certains patients et se manifester par la reprise de la symptomatologie clinique initiale ou par l'apparition de symptômes nouveaux. Toutefois les complications vasculaires graves liées à une artérite active demeurent exceptionnelles après le 1^er^ trimestre, chez un patient correctement traité.

## Conclusion

La maladie de Horton intéresse dans la majorité des cas de nombreux organes. Les atteintes oto-rhino-laryngologiques restent mal connues par nos spécialistes. La collaboration entre rhumatologue, interniste, stomatologue et ORL est essentielle. La symptomatologie ORL de la Maladie de Horton est multiple et peut constituer une aide au diagnostic, elle peut être révélatrice de la maladie pour autant que l'on connaisse bien le polymorphisme de la maladie, elle est généralement significative lors de sa rechute; Ces données s'accordent avec l'adage selon lequel la maladie de Horton doit être un diagnostic quotidiennement a l'esprit du médecin amené à suivre les personnes âgées [10].

## References

[1] Mahfoudi M, Mamlouk H, Turki S, Kheder A (2015). Maladie de Horton révélée par une dyspnée. The Pan African Médical Journal..

[2] Caignard A, Milea D (2013). Complications ophtalmologiques de la maladie de Horton: La Revue du Praticien Médecine Générale.

[3] Kassen H, ElGharbi T, Hamadi K, Dresco E, Turner L (2011). Toux chronique relevant une maladie de Horton. Rev Med Interne..

[4] Mrani A, Souirti Z, Belahsen MF, Messouak O (2012). Syndrome confusionnel révélant la maladie de Horton. Revue Neurologique..

[5] Liozon E, Ly K (2008). Maladie de Horton. Rev Prat..

[6] Harton PY, Lambert M (2009). La maladie de Horton. Médecine Nucléaire..

[7] Liozon E (2006). Maladie de Horton La Revue du Praticien. Médecine Générale.

[8] Liozon E, Nadalon S, Longuet O, Loustaud V, Soria P, Ly K, Vidal E (2002). Les manifestations stomatologiques et ORL de la maladie de Horton: étude prospective de 214 malades. Rev Méd Interne..

[9] Barrier J (2002). Maladie de Horton et pseudopolyarthrite rhizomelique. Rev Prat..

[10] Letellier P, Andres L, Dassonville I, Gires C, Zoulim A, Commpére JF, Babin E, Rousselot P (2000). Les manifestations ORL et stomatologiques de la maladie de Horton: Étude prospective de 1980 à 2000 portant sur 250 patients. Rev Méd Interne..

[11] Liozon E (2005). La maladie de Horton. Rev Prat..

[12] Frachet B (2004). Acouphènes subjectifs La Revue du Praticien-Médecine Générale.

[13] Becourt-Verlomme C, Barouky R, Alexandre C, Gonthier R, Laurent H, Vital Durand D, Rousset H (2001). Symptômes inauguraux de la maladie de Horton sur une série de 260 patients. Rev Med Interne..

[14] Kelkel E, Sarrot-Reynauld F, Dussud C, Pasquier D, Massot C (1991). Le retard diagnostique dans la maladie de Horton Analyse du délai diagnostique à partir d'une étude rétrospective de 130 cas. Rev Med Interne..

